# A cross-sectional analysis of prescription and stakeholder surveys following essential medicine reform in Guangdong Province, China

**DOI:** 10.1186/s12913-015-0778-3

**Published:** 2015-03-13

**Authors:** Wen-yuan Zhang, Ying-ran Li, Yun-jing Li, Xue-qin Li, Wei-guo Zhao, Rong-zhi Lu

**Affiliations:** Department of Pharmacy, Zhongshan Hospital of Sun Yat-sen University, Zhongshan, 528403 China; School of Pharmaceutical Sciences, Sun Yat-sen University, Guangzhou, 510006 China

**Keywords:** Essential medicine, Prescription analysis, China, Rational use of medicines, Zero mark-up policy

## Abstract

**Background:**

An essential medicine (EM) system has been implemented in China to reduce patients’ financial burden and to make the use of drugs more rational. This study aims to evaluate the current state of the EM system in Guangdong Province.

**Methods:**

We conducted surveys in 21 cities in 2012, covering 98 medical institutions, 1,509 doctors, 17 medicine manufacturers, and 17 distribution companies. We also reviewed outpatient prescriptions (n = 9,941) for treating hypertension, diabetes, bacterial infections and gout to measure the rational use of drugs in secondary and tertiary (upper-level) hospitals.

**Results:**

The percentage of non-priority EM use ranged from 8.1% to 10.7% in upper-level hospitals, and this non-priority use significantly increased prescription drug costs. Other types of inappropriate medicine use were found more frequently in treating bacterial infections (7.4%) than in treating hypertension (1.6%), diabetes (1.3%) and gout (1.7%). Tertiary hospitals prescribed fewer EMs than secondary hospitals; moreover, tertiary hospitals had higher prescription drug costs. The zero mark-up policy decreased prescription drug costs in secondary hospitals. The survey revealed that forced full-prescription EM use might lead to fewer patient visits to primary hospitals. Manufacturers had halted the production of four (1, 23) types of EMs at the time of the survey.

**Conclusions:**

Encouraging the priority use of EMs and implementation of the zero mark-up policy were effective in curtailing prescription medicine costs in upper-level hospitals. Further work should focus on the following: creating guidelines to enhance rational prescription behavior, establishing policies to support EM use in upper-level hospitals and improving the bidding system to ensure a steady supply of the lowest-priced generic drugs.

## Background

Health care expenditures per capita have risen dramatically in China – from US$ 21 to US$ 220 between 1995 and 2010 [1]. As a result of this rapid rise in health care expenditures, more and more Chinese citizens are complaining about the high cost of medical care and the inequitable distribution of medical resources. Health care reform was launched in 2009 to achieve universal health care coverage and reduce the financial burden on patients [2]. The details of this reform include building a national list of essential medicines (EMs), centralizing medical procurement and provincial-level tender, and providing EMs at cost in primary hospitals (the zero mark-up policy). This reform also set clinical standards aimed at eradicating irrational prescription behavior [3], such as doctors overprescribing drugs to receive kickbacks from pharmaceutical salesmen. The study showed that implementing an EM system was effective in shifting prescribing indicators toward the standard values recommended by the WHO [4].

The health care reform affects the interests of the entire chain of medical services. Many health care providers have complained about higher workloads and lower incomes since the reform [5]. Additionally, some primary hospitals have found alternative ways to compensate for the losses caused by the reform and when government subsidies are insufficient [6]. Primary hospitals are asked to prescribe only EMs and to adopt the zero mark-up policy. The current reform focuses on primary hospitals, and many papers have reported the effects of the reform on practices at primary hospitals in remote areas [7-9] and in individual cities [10-12]. Meanwhile, secondary and tertiary (upper-level) hospitals are asked to prescribe EMs as a priority and encouraged to pilot the zero mark-up policy. Although these upper-level hospitals treat the majority of patients in China, insufficient attention has been paid to them since the reform.

In the present study, we reviewed prescriptions (n = 9,941) in upper-level hospitals for treating hypertension, diabetes, bacterial infections and gout. Furthermore, we integrated surveys of different levels of hospitals and doctors, pharmaceutical manufacturers and distribution companies to systematically evaluate the current state of the EM system in one of China’s most developed provinces (Guangdong).

## Methods

Guangdong Province is located in southern China; it includes 21 cities and has a population of 104.3 million. Guangdong is the largest province in China as measured by GDP, which reached approximately $904.05 billion in 2012. This study used the “2009 National Essential Medicines List,” which contained 307 generic drugs, and the “Guangdong Provincial Supplementary Essential Medicines List,” which included another 244 essential drugs. We intended to enroll one secondary, one tertiary and four primary hospitals in each city to participate in the study. Ultimately, 35 upper-level and 63 primary hospitals participated in the survey, and 29 upper-level hospitals participated in the prescription analysis.

Semi-structured questionnaires were designed for this study based on a literature review and a consultation with a health care specialist and were approved by the provincial and local health bureaus. A regional director was set in each city to ensure the study’s quality, and each director was an experienced clinical pharmacist. The directors were responsible for the survey and data monitoring. This study was approved by the ethics committees at Zhongshan Hospital of Sun Yat-sen University. Participants could not be identified from the recorded data, and all participants gave their informed consent. The final results would be submitted to the provincial health bureaus. The study included two parts: a cross-sectional analysis of prescription and semi-structured surveys.

### Prescription analysis

Outpatient prescriptions were analyzed in each hospital in June 2012 under the following official sets of guidelines: “Practices of hospital prescription review”, “Guiding principles of the clinical use of antibiotics”, and “Guiding principles of prescription review in Guangdong Province”. Prescriptions for treating hypertension, diabetes, bacterial infections and gout were selected. This analysis sought to characterize the overall rate of prescribing EMs and the rate of inappropriate prescribing practices. In each facility, 100 prescriptions for each disease were systematically selected by their clinical pharmacist and regional director using an arithmetic method. If fewer than 100 prescriptions were issued, then all prescriptions were included; if there was any discrepancy with respect to whether a prescription constituted an irrational prescription, then a consensus was reached by discussion. The number of total drugs, the number of EMs, the cost of all drugs, the cost of the EMs, and the inappropriate use of medicines were recorded for this study. The data were subjected to stratified analysis based on the hospital type, the rational use of medicines and whether the zero mark-up policy was adopted.

### Surveys of health care facilities, manufacturers and distribution companies

In each health care facility, data on EM usage in 2011 was collected according to official documents. A semi-structured questionnaire was then completed by the chief pharmacy manager to determine the reform’s impact on each facility. Meanwhile, semi-structured questionnaires were distributed to doctors. At least 16 doctors were chosen in four randomly selected medical departments in each upper-level hospital, and 10 doctors were chosen in each primary hospital. These doctors were asked questions about EM prescription. We also chose large medicine manufacturers (n = 17) and distribution companies (n = 17) in Guangdong Province for the investigation. Chief executives were requested to complete the semi-structured questionnaires. The questionnaire included questions about their general circumstances, their suggestions and their concerns regarding the current reform.

### Analysis method

All the data collected were carefully entered into a spreadsheet created in Excel 2010 (Microsoft, USA). The data were then transferred to SPSS (version 18.0) for statistical analysis. A group comparison was performed using the Mann–Whitney U test or the chi-square test. A Spearman’s correlation test was employed to determine the relationship between the ratio of EMs in each prescription and prescription drug costs. The level of significance was set at P ≤ 0.05. The data in this paper reflect median values (25th–75th interquartile range).

## Results

### Prescription analysis

At the deadline, 25 hospitals had fully reported their prescription analyses of the four diseases, and four hospitals had partially reported their records. A total of 10,117 prescription records were reported, and 176 of these were rejected because of missing information or input errors. Ultimately, 9,941 (98.2%) prescriptions were included in the analysis.

### Rational use of medicines

The WHO defines the rational use of medicines as occurring when “patients receive medications appropriate to their clinical needs, in doses that meet their own individual requirements, for an adequate period of time, and at the lowest cost to them and their community.” The irrational use of medicines refers to medical use without following these criteria. We identified the following common types of irrational medicine use: drug use without indicators, inappropriate drug selection, inappropriate dosage or usage, and inappropriate drug combination. The percentage of non-priority use of EMs ranged from 8.1% to 10.7% (Table 1) in upper-level hospitals. Non-priority use of EMs significantly increased the prescription drug costs of treating hypertension, gout and bacterial infections compared with the rational use of medicines. Other types of irrational medicine use were found more frequently for bacterial infections (7.4%) than for hypertension (1.6%), diabetes (1.3%) and gout (1.7%).

**Table 1:** **Rational drug use in treating hypertension, diabetes, gout and bacterial infections**

**Disease type (n)**	**Non-priority use of EMs (Rx cost; RMB)**	**Rational drug use (Rx cost; RMB)**	**% Non-priority use of EMs (n)**	**% Other irrational use (n)**
**Hypertension**	211.2	132.4**	9.1%	1.6%
(2445)	(114.1-335.3)	(71.9-252.3)	(222)	(38)
**Diabetes**	197.7	190.6	8.1%	1.3%
(2850)	(95.9-370.5)	(95.2-322.7)	(231)	(38)
**Gout**	90.1	60.2**	10.7%	1.7%
(2075)	(36.0-206.3)	(27.9-118.3)	(221)	(36)
**Bacterial infections**	100.5	89.1*	10.7%	7.4%
(2571)	(64.0-151.2)	(52.2-153.7)	(274)	(189)

Rx = prescription; EMs = essential medicines.

On June 1, 2012, at the beginning of the survey, the conversion rate was RMB 6.33 to USD 1.00. Data are shown as medians (25th–75th interquartile range). *p < 0.05 and **p < 0.01 compared with the non-priority use of EMs.

### Prescription pattern in upper-level hospitals

The prescription pattern in Table 2 shows that tertiary hospitals prescribed fewer EMs than secondary hospitals and with higher median prescription drug costs. The total number of drugs showed no significant difference with respect to prescription of medicines for hypertension, diabetes and gout, but the number was lower for prescriptions for bacterial infections in tertiary hospitals (mean ± SD: 3.51 ± 1.44 for secondary and 2.92 ± 1.53 for tertiary hospitals). The Spearman’s correlation coefficient for the ratio of EMs to prescription drug costs was −0.344 for hypertension, −0.200 for diabetes, −0.282 for bacterial infections, and −0.477 for gout in upper-level hospitals. These correlations were significant but not strong (p < 0.001).

**Table 2:** **A comparison of secondary and tertiary hospitals regarding the availability, number and cost of essential medicines**

**Hospital (n Rx)**	**EMs/Rx number (%)**	**EM number**	**Total drug number**	**EMs/Rx cost (%)**	**EM cost (RMB)**	**Total Rx cost (RMB)**
**Hypertension (r = −0.344)#**						
Secondary	80	2	3	73	44.7	98.1
(1058)	(50–100)	(1–3)	(2–4)	(32–100)	(15.9-92.7)	(42.9-178.22)
Tertiary	50**	1**	3	34**	51.24	184.9**
(1387)	(25–75)	(1–2)	(2–4)	(4–82)	(7.1-133.5)	(98.6-329.2)
**Diabetes (r = −0.200)**						
Secondary	80	2	3	91	74.9	128.8
(1164)	(50–100)	(1–3)	(2–4)	(41–100)	(24.6-167.2)	(63.9-245.3)
Tertiary	50**	1**	2	53**	100.1*	231.1**
(1686)	(25–100)	(1–2)	(1–4)	(8–100)	(14.9-212.4)	(133.9-384.9)
**Gout (r = −0.477)**						
Secondary	75	2	3	77	17.7	35.6
(956)	(50–100)	(1–3)	(2–4)	(21–100)	(5.2-30.2)	(20.9-68.3)
Tertiary	40**	1**	3	5**	4.0**	99.2**
(1119)	(0–60)	(0–2)	(2–4)	(0–24)	(0–19.9)	(50.3-182.0)
**Bacterial infections (r = −0.282)**						
Secondary	60	2	3	47	31.2	85.7
(1196)	(40–80)	(1–3)	(2–5)	(13–92)	(9.8-66.9)	(51.85-131.4)
Tertiary	50**	1**	3**	30**	22.4**	99.8**
(1375)	(20–80)	(1–2)	(2–4)	(1–97)	(0.8-62.3)	(55.9-180.1)

Rx = prescription; EMs = essential medicines.#Spearman correlation coefficient between the ratio of EMs and prescription medicine costs in upper-level hospitals (p < 0.001).

On June 1, 2012, at the beginning of the survey, the conversion rate was RMB 6.33 to USD 1.00. Data are shown as medians (25th–75th interquartile range). *p < 0.05 and **p < 0.01 compared with secondary hospitals.

### Zero mark-up policy in secondary hospitals

In the secondary hospitals involved in the prescription analysis, three had adopted the zero mark-up policy (Table 3). The results indicated that the zero mark-up policy decreased the total medicine costs and EM prescription costs of treating most diseases. The hospitals that had adopted the zero mark-up policy prescribed fewer drugs for hypertension (mean ± SD: 2.01 ± 1.18 for those with zero mark-up and 2.36 ± 1.43 for those without zero mark-up) and bacterial infections (mean ± SD: 3.17 ± 1.37 for those with zero mark-up and 3.62 ± 1.44 for those without zero mark-up) but prescribed more drugs for gout (mean ± SD: 3.39 ± 1.37 for those with zero mark-up and 2.81 ± 1.31 for those without zero mark-up).

**Table 3:** **The comparison of the zero mark-up policy in secondary hospitals**

**Zero mark-up**	**EMs/Rx number (%)**	**EM number**	**Total drug number**	**EMs/Rx cost (%)**	**EM cost (RMB)**	**Total Rx cost (RMB)**
**Hypertension**						
Adopted	100	2	2	100	30.2	65.5
(299)	(60–100)	(1–3)	(2–3)	(39–100)	(14.0-83.3)	(17.6-140.3)
Not adopted	75**	2**	3**	64**	51.3**	102.1**
(759)	(50–100)	(1–3)	(2–5)	(26–100)	(20.0-97.6)	(54.1-200.8)
**Diabetes**						
Adopted	67	2	3	56	24.5	100.5
(304)	(50–100)	(1–3)	(2–3)	(13–100)	(9.6-105.3)	(39.9-189.3)
Not adopted	100**	2	3	99**	85.4**	136.0**
(860)	(50–100)	(1–3)	(1–4)	(52–100)	(38.7-180.9)	(69.5-261.1)
**Gout**						
Adopted	75	3	3	66	11.6	30.5
(232)	(50–100)	(1–4)	(3–5)	(14–100)	(4.3-25.3)	(13.8-82.1)
Not adopted	75	2**	3**	84	21.0**	35.9
(724)	(50–100)	(1–4)	(2–4)	(21–100)	(5.2-31.5)	(21.4-62.1)
**Bacterial infections**						
Adopted	60	2	3	55	32.6	76.0
(297)	(30–100)	(1–3)	(2–4)	(8–100)	(3.6-71.5)	(42.0-117.9)
Not adopted	60	2**	3**	43	31.0	88.6**
(899)	(40–80)	(1–3)	(3–5)	(14–88)	(12.2-65.3)	(53.2-139.4)

Rx = prescription; EMs = essential medicines.

On June 1, 2012, at the beginning of the survey, the conversion rate was RMB 6.33 to USD 1.00. Data are shown as medians (25th–75th interquartile range). **p < 0.01 compared with the zero mark-up group.

### Surveys in health care facilities

The EM usage for 2011 is shown in Table 4 for 63 primary, 16 secondary, and 19 tertiary hospitals. The stored types of EMs constituted 97.5% (90.0%, 100.0%), 37.5% (30.0%, 40.0%), and 29.9% (28.5%, 35.3%) of the total types of drugs stored by primary, secondary, and tertiary hospitals, respectively. Furthermore, 92% of primary hospitals, 25% of secondary hospitals, and 5% of tertiary hospitals had adopted the zero mark-up policy.

**Table 4:** **Essential medicine usage in hospitals in Guangdong Province in 2011**

**Hospital (n)**	**EMs types**	**EMs/Total types (%)**	**EMs/Total sales (%)**	**Outpatient EM reimbursement rate (%)**	**Inpatient EM reimbursement rate (%)**	**Adopted zero mark-up (%)**
Primary	305	97.5	95.7	70.0	86.5	92%
(63)	(239–425)	(90.0-100.0)	(84.8-100.0)	(50.0-91.0)	(75.0-100.0)	
Secondary	345	37.5	23.2	75.0	82.5	25%
(16)	(298–379)	(30.0-40.0)	(16.9-25.6)	(65.0-100.0)	(50.0-98.0)	
Tertiary	377	29.9	15.0	59.5	87.5	5%
(19)	(345–422)	(28.5-35.3)	(12.1-21.0)	(41.7-75.2)	(59.0-100.0)	

EMs = essential medicines. Data are shown as medians (25th–75th interquartile range).

After the reform, the change in the number of patient visits was significantly associated with hospital levels, χ^2^ (2, N = 98) = 30.16, p < 0.001, Cramer’s V = 0.56. As reported by the person in change in each hospital, the number of patients decreased in 54.0% of primary hospitals versus 2.9% of upper-level hospitals, increased in 22.2% of primary hospitals and 22.9% of upper-level hospitals, and remained unchanged in 23.8% of primary and 74.3% of upper-level hospitals after the reform. Moreover, 47.6% of primary hospitals believed that government subsidies were not sufficient to compensate for the losses from zero mark-up, 15.9% believed that the subsidies were sufficient, and 36.5% believed that the subsidies compensated for slightly less than the loss. The questionnaire results showed that some local health authorities created a regional EM list.

### Surveys of doctors

A total of 1,600 questionnaires were distributed to doctors in these hospitals, and 1,509 valid questionnaires were obtained (650 from primary hospitals and 859 from upper-level hospitals), yielding a valid response rate of 94.3%. Only 8.6% of doctors in primary hospitals and 17.7% in upper-level hospitals believed that the EMs fully met clinical needs. Furthermore, many doctors in primary hospitals reported that insufficient types of EM hindered their treatment. We also investigated the reasons for not prescribing EMs in upper-level hospitals, and the results indicate the following reasons: 48.8% for “I do not trust the quality and efficacy of EMs”; 35.5% for “The drugs on the list are not enough for clinical use”; 10.2% for “The patients refuse to use EMs”; and 5.5% “I do not know which drugs are the EMs”.

### Surveys of manufacturers and distribution companies

The annual output value was 393 (228, 1488) million Yuan for these manufacturers, and 64 (43, 96) types of drugs were produced, in which EMs accounted for 40% (20%, 60%). There were 4 (1, 23) types of EMs for which production had been halted at the time of the survey. In distribution companies, the four factors cited as most influential to distribution were delayed payment (35%), low demand (12%), high distribution costs (23%), and cessation of production by manufacturers (29%). The average period of payment settlement was 64% between 3 and 6 months, 29% at more than 6 month, and only 6% within the contracted period of 2 months.

## Discussion

This investigation revealed the EM prescription pattern and the current state of the EM system in Guangdong Province. Two effective ways to reduce prescription drug costs in upper-level hospitals include ensuring priority EM use and adopting the zero mark-up policy. However, it is unrealistic to promote the zero mark-up policy in upper-level hospitals because of limited government budgets. Improving the priority use of essential drugs is a more realistic approach. Currently, the government encourages hospitals to recruit enough clinical pharmacists to monitor prescriptions because clinical pharmacists are thought to positively influence the appropriate use of prescription drugs [13].

Although the priority use of EMs is encouraged, their use is not mandatory in upper-level hospitals. We found that the ratio of non-priority use of EMs was not high, but the use of non-priority EMs significantly increased prescription medicine costs. Except for non-priority EM use, the most common inappropriate use was to treat bacterial infections. In 2009, the Ministry of Health published a strict policy to strengthen the management of antibiotics, but inappropriate antibiotic use still accounted for a large proportion of irrational drug use. The misuse of antibiotics poses considerable risks for drug resistance, toxicity, and allergic reactions. Multi-faceted strategies are required to improve rational antibiotic use, including education, replacement of economic incentives, promotion of best practices, and investment in surveillance [14].

Tertiary hospitals prescribed fewer EMs than secondary hospitals and had higher prescription drug costs, which might be partly explained by the fact that the tertiary hospitals typically provided treatment for costly and complex diseases [15]. Except for the treatment of bacterial infections, the average number of drugs per prescription showed no significant difference between secondary and tertiary hospitals. A median of three drugs were used per prescription, which was markedly higher than the WHO recommended values of 1.3 to 2.0 [7]. The reason for this discrepancy may be attributable to symptomatic treatment and inappropriate practice patterns by doctors.

Consistent with previous studies conducted in primary hospitals [5], the secondary hospitals that had adopted the zero mark-up policy also decreased total prescription costs. However, we did not find that implementing the zero mark-up policy would necessarily decrease the prescription drug numbers or increase the use of EMs. Other researchers have found that the number of drugs prescribed was reduced but that the number of injectable drugs increased in primary hospitals [5,16]. One aim of the zero mark-up policy is to improve the rational use of medicines by eliminating economic incentives for health care institutions, but current studies indicate that this objective has not been reached [17,18].

Improvements in primary care are critical because they not only can decrease health care expenditures per capita but also can boost health conditions and health care outcomes [19,20]. However, this survey found that the number of patient visits to most primary hospitals decreased. Other studies have reported the same phenomenon: because the EMs did not fully meet clinical needs, some patients were transferred to upper-level hospitals [21]. The sufficiency of the EM supply was also a concern for doctors in primary hospitals in this survey. This phenomenon has attracted government attention, and drug numbers increased in a new national EM list published in September 2012 [22]. However, it remains unresolved whether the new list can overcome the effects of prescription restrictions in primary hospitals. Meanwhile, the zero markup policy made grassroots hospitals rely more heavily on financial subsidies from local governments [5,23]. However, it was reported that local governments may not take sufficient responsibility for the scientific design and funding of the EM policy, which could threaten the viability of the zero mark-up policy [6].

It is critical to maintain a balance between drug price and quality in the bidding system. Overweighting the price in the bidding system would result in the production of low-quality EMs or make manufacturers reluctant to produce profitless EMs [21]. Currently, the availability of lowest-priced generic drugs in China has become a substantial problem [24]. To promote normal production and supply of low-cost drugs, the “Guangdong Provincial Low-cost Drug List of EMs” has been created. The list of drugs will only follow the quality standards [25]. The survey showed that payments from hospitals were always delayed. To improve timely payment settlement, a centralized settlement system will be established by the Guangdong Pharmaceutical Exchange that will automatically settle accounts according to a “Settlement Services Agreement” when the settlement period is longer than 60 days [26].

The present research has several limitations. The findings in Guangdong Province may not be regarded as representative of all of China. The irrational use of medicines was manually assessed, which might indicate that there is potential bias. In addition, only a few secondary hospitals had adopted the zero mark-up policy, which limited the sample size.

## Conclusions

Two effective ways to reduce prescription drug costs in upper-level hospitals are to ensure priority EM use and to adopt the zero mark-up policy. Further work should be focused on improving EM use in upper-level hospitals. For primary hospitals, a more rational financial subsidy policy should be offered to ensure the viability of zero mark-up policies. Doctors should receive guidance regarding rational prescribing practices, particularly in treating bacterial infections. For manufacturers, a scientific design in the bidding system should be implemented to ensure a steady supply of the lowest-priced generic drugs. For distribution companies, timely settlement should be improved.
